# Low Vitamin D Status Is Associated with Nonalcoholic Fatty Liver Disease Independent of Visceral Obesity in Korean Adults

**DOI:** 10.1371/journal.pone.0075197

**Published:** 2013-10-09

**Authors:** Ji A. Seo, Chai Ryoung Eun, Hyunjoo Cho, Seung Ku Lee, Hye Jin Yoo, Sin Gon Kim, Kyung Mook Choi, Sei Hyun Baik, Dong Seop Choi, Hyung Joon Yim, Chol Shin, Nan Hee Kim

**Affiliations:** 1 Division of Endocrinology, Department of Internal Medicine, Korea University College of Medicine, Seoul, Korea; 2 Division of Pulmonary and Critical Care Medicine, Department of Internal Medicine, Korea University College of Medicine, Seoul, Korea; 3 Division of Hepatology, Department of Internal Medicine, Korea University College of Medicine, Seoul, Korea; Institute of Medical Research A Lanari-IDIM, University of Buenos Aires-National Council of Scientific and Technological Research (CONICET), Argentina

## Abstract

**Objective:**

To investigate the association between serum 25-hydroxyvitamin D [25(OH)D] levels and nonalcoholic fatty liver disease (NAFLD) independent of visceral obesity in Koreans and to examine whether the associations differ according to the presence of diabetes or insulin resistance.

**Research Design and Methods:**

A total of 1081 adults were enrolled from a population-based cohort in Ansan city. Serum 25(OH)D concentrations were measured in all subjects. Insulin resistance was measured by homeostasis model assessment of insulin resistance (HOMA-IR). Using computed tomography, NAFLD was diagnosed if the liver attenuation index (LAI, the difference between the mean hepatic and splenic attenuation) was <5 Hounsfield Units.

**Results:**

In subjects with diabetes (n = 282), 25(OH)D levels were negatively associated with waist circumference, fasting insulin, HOMA-IR, triglyceride levels, and visceral abdominal fat, and were positively associated with LAI after adjusting for age, sex, season, exercise, and vitamin supplementation. In subjects without diabetes, only triglyceride level was negatively associated with 25(OH)D. The adjusted odds ratio (OR) for NAFLD increased sequentially across decreasing quartiles of 25(OH)D in subjects with diabetes even after adjusting for visceral fat [Q1 vs. Q4; OR for NAFLD 2.5 (95% CI:1.0–6.2)]. In contrast, no significant difference in OR was observed in subjects without diabetes. When we classified non-diabetic subjects by HOMA-IR, an increase in the OR for NAFLD across decreasing quartiles of 25(OH)D was observed in the high HOMA-IR (≥2.5) group [n = 207, Q1 vs. Q4; OR 3.8(1.4–10.3)], but not in the low HOMA-IR (<2.5) group [n = 592, OR 0.8 (0.3–1.9)].

**Conclusions:**

Low vitamin D status is closely associated with NAFLD, independent of visceral obesity in subjects with diabetes or insulin resistance.

## Introduction

Nonalcoholic fatty liver disease (NAFLD) is one of the most frequent causes of abnormal liver function and correlates with central adiposity, insulin resistance, and type 2 diabetes mellitus [1], [2], [3]. Accumulating evidence suggests that altered vitamin D homeostasis is associated with chronic liver diseases, including NAFLD and chronic hepatitis C [4], [5], [6]. In patients with biopsy-proven NAFLD, serum 25-hydroxyvitamin D [25(OH)D] levels were lower than in healthy controls and 25(OH)D levels were correlated with histological severity of NAFLD [6]. In addition, in a diet-induced steatohepatitis rat model, phototherapy or active vitamin D (1α-hydroxy-cholecalciferol) treatment ameliorated the progression of nonalcoholic steatohepatitis [7]. However, most previous studies were either based on experimental models or had a small sample size of human participants.

Several studies have suggested that vitamin D deficiency and NAFLD are commonly associated with central obesity and insulin resistance [2], [8], [9]. Therefore, central obesity can be a linking mechanism of hypovitaminosis D and NAFLD. Nevertheless, it is not known whether central obesity mediates the association of vitamin D deficiency with NAFLD.

There is also evidence that the impacts of vitamin D deficiency on metabolic disturbances are accentuated in subjects with type 2 diabetes compared with non-diabetic controls [10]. In addition, vitamin-D deficient rats fed a westernized diet which could induce insulin resistance had more pronounced features of NAFLD and hepatic necroinflammation than vitamin-D deficient rats fed a low-fat diet, which does not induce insulin resistance [11]. Furthermore, meta-analysis of randomized controlled trials revealed no benefit from vitamin D supplementation in patients with normal glucose tolerance, but did show an improvement in fasting glucose and insulin resistance in patients with impaired glucose tolerance or insulin resistance [12]. These studies suggest that the clinical implications of vitamin D deficiency may vary according to the status of insulin resistance or diabetes. However, this hypothesis has not yet been proven.

The aim of the present study was to investigate the relationship between serum 25(OH)D concentrations and NAFLD in a large number of subjects in a population-based cohort after controlling for visceral obesity. Furthermore, we examined whether the associations differ according to the presence of diabetes or insulin resistance.

## Methods

### Study Subjects

All study subjects were derived from the Ansan cohort of the Korean Genome Epidemiology Study (KoGES), an ongoing population-based cohort study that began in 2001. Details of the study design and sampling method have been described in previous reports [13], [14]. Briefly, the cohort consisted of 2,523 men and 2,497 women aged 40–69 years at baseline who had undergone a comprehensive health examination, including interviews. They were followed up biennially. Among the 3,262 subjects who participated in the fifth biennial examination, we included 1,307 subjects (515 men, 792 women) who had their serum 25(OH)D levels and visceral fat mass measured by computed tomography from May 11^th^, 2009 to January 8^th^, 2010. To be eligible for the study, subjects had to fulfill the following criteria: no evidence of viral hepatitis [HBsAg(+) or anti-HCV Ab(+)], no history of current or past excessive alcohol drinking as defined by an average ≥140 g/week of alcohol, and absence of history of hemochromatosis, autoimmune hepatitis, cirrhosis, or other chronic liver disease. After excluding subjects who had missing data for either alcohol consumption, viral markers, or a diagnosis of diabetes (n = 18), a total of 1,081 subjects (345 men, 736 women) over 45 years of age were included in this study. When we compared the characteristics of subjects who were enrolled in the present study with those who were excluded (the remainder) among the participants of the fifth biennial examination, both groups had similar clinical characteristics. However, study subjects had higher fasting insulin and lower HDL-cholesterol levels than the remainder (Table S1). Subjects were asked in interviews whether, during the previous year, they had used vitamin supplements (multivitamin and/or vitamin D), medications for diabetes, hypertension, or dyslipidemia, or had a previous history of cardiovascular diseases including angina, stroke or myocardial infarction, cancer, pulmonary diseases, or recent hospitalization. Exercise status was categorized as no exercise, light exercise (<3 times/week), or regular exercise (≥3 times/week, ≥30 minutes per session) during the previous month. Each participant signed an informed consent form. This study was performed according to the principles of the Declaration of Helsinki of the World Medical Association, and was approved by the institutional review committee at Korea University Ansan Hospital.

### Anthropometric Measurements and Blood Tests

Anthropometric measurements for each participant were taken after an overnight fast while the subject wore light clothing and no shoes. Height was determined using a fixed wall-scale measuring device and was measured to the nearest 0.1 cm. Weight was measured to the nearest 0.1 kg using an electronic scale that was calibrated prior to each measurement. Body mass index was calculated as weight in kilograms divided by the square of height in meters. Waist circumference was measured to the nearest 0.5 cm in a horizontal plane at the level of the umbilicus at the end of normal expiration. Blood pressure was measured by trained technicians using a mercury sphygmomanometer (Baumanometer; W. A. Baum, Copiague, NY, USA). Blood pressure measurements were taken with the subject in the supine position after at least a 10-min rest period. Measurements were recorded to the nearest 2 mmHg. Blood samples were taken in the morning after an overnight fast. All subjects underwent a 75 g oral glucose tolerance test (OGTT). We measured fasting and post two-hour OGTT-glucose and insulin levels. Plasma glucose, serum triglycerides, total-cholesterol, HDL-cholesterol, alanine transferase (ALT) and aspartate transferase (AST) levels were measured using an autoanalyzer (ADVIA1650; Siemens, NY, USA). Insulin was measured with an immunoradiometric assay kit (INS-IRMA kit; Biosource, Nivelles, Belgium) using a gamma counter system (Packard, USA). Insulin resistance was estimated using homeostasis model of assessment (HOMA-IR), and was calculated as fasting glucose (mmol/L) x fasting insulin (uU/mL)/22.5 [15]. Serum 25(OH)D concentrations were measured using a chemiluminescent immunoassay (CLIA) kit (DiaSorin, Stillwater, OK, USA) in duplicate with the LIAISON analyzer (DiaSorin, Dietzenbach, Germany). The DiaSorin kit detected both 25(OH)D_3_ and 25(OH)D_2_ in total. The inter-assay coefficient of variation for 25(OH)D was 6%.

### Diagnosis of Nonalcoholic Fatty Liver Disease and Measurement of Visceral Fat Using Computed Tomography

Abdominal adipose tissue areas were quantified by a single slice computed tomography (CT) scan at 120 kV of exposure (Brilliance 64; Philips, Cleveland, OH, USA). A 5 mm CT slice scan was acquired at the L4–L5 vertebral interspace to measure visceral abdominal fat areas by measuring the mean value of all pixels within the range of −190 to −30 Hounsfield units. The images were converted into files compatible with a commercial software program (EBW; Philips, Cleveland, OH, USA).

The hepatic attenuation was measured by means of random selection of three circular regions (1 cm^2^) of interest (ROI) on three transverse sections. Those transverse sections were selected at T11 mid-body, T11–T12 interspace, and T12 mid-body levels. To provide an internal control, the mean splenic attenuation was also calculated by averaging every two random ROI splenic attenuation measurement values on each of the three transverse sections used for the evaluation of hepatic attenuation. The liver attenuation index (LAI), derived from the difference between the mean hepatic and splenic attenuation, was used as a parameter for the diagnosis of non-alcoholic fatty liver disease (NAFLD). A LAI of <5 Hounsfield Units (HU) is a known predictor of >5% steatosis [16], and histological confirmation of NAFLD requires a minimum of 5% steatosis [17]. Therefore, NAFLD was defined when the LAI value was <5 HU in this study.

### Diagnosis of Diabetes and Insulin Resistance

A diagnosis of diabetes was made by one of the following criteria: 1) fasting plasma glucose ≥7.0 mmol/L; 2) post two-hour OGTT-glucose ≥11.1 mmol/L; 3) a previous history of medication for diabetes, or a previous diagnosis of diabetes. Non-diabetic subjects were classified into insulin resistant (IR+) and non-insulin resistant (IR−) groups using a HOMA-IR cutoff point of the 75th percentile [18]. The cutoff point of the 75th HOMA percentile in non-diabetic men and women was 2.56 and 2.52, respectively.

### Statistical Analysis

Statistical analyses were conducted using SAS version 9.1 for Windows (SAS Institute Inc., Cary, NC, USA). Continuous variables are expressed as means and standard deviations or median and interquartile ranges if their distributions are skewed (Table 1). For continuous variables, the t-test was used to assess differences in means according to the status of diabetes or insulin resistance. 25(OH)D, insulin, HOMA-IR, triglyceride, ALT, and AST levels were log-transformed due to skewed distributions and *p* values were from the t-test using log-transformed values (Table 1). For categorical data, the chi-squared test was used to assess differences in proportions across the categories.

**Table 1:** Characteristics of study subjects according to the presence or absence of diabetes.

Variables	Non-DM (n = 799)	DM (n = 282)	*p-value*
Age (years)	55.8±6.7	59.8±8.2	<.0001
Men, n (%)	240 (30.0)	104 (36.9)	0.034
Waist circumference (cm)	79.4±7.8	83.6±8.5	<.0001
Body mass index (kg/m^2^)	24.4±2.8	25.3±3.1	<.0001
Systolic blood pressure (mmHg)	112.9±14.4	115.7±15.3	0.006
Fasting plasma glucose (mmol/L)	5.1±0.4	6.9±2.9	<.0001
Fasting insulin* (uU/mL)	8.5 (6.6, 10.9)	10.7 (8.1, 14.8)	<.0001
HOMA-IR*	1.9 (1.5, 2.5)	2.9 (2.1, 4.3)	<.0001
Triglycerides* (mmol/L)	1.3 (1.0, 1.9)	1.6 (1.1, 2.2)	<.0001
HDL-cholesterol (mmol/L)	1.1±0.3	1.0±0.2	<.0001
ALT*(IU/L)	20 (16, 26)	22 (18, 31)	<.0001
AST*(IU/L)	23 (21, 27)	24 (21, 28)	0.004
25(OH)D* (nmol/L)	34.2 (24.7, 46.9)	35.4 (24.4, 48.1)	0.865
Visceral abdominal fat (cm^2^)	77.3±33.5	97.7±42.4	<.0001
Liver attenuation index (HU)	11.8±9.7	7.1±10.8	<.0001
NAFLD [LAI<5, n (%)]	131 (16.4)	98 (34.8)	<.0001
Vitamin D deficiency [25(OH)D<50 nmol/L, n (%)]	638 (79.8)	218 (77.3)	0.366
Duration of diabetes (years)		7.35±4.7	
Diabetes medication, n (%)		99 (35.1)	
Hypertension, n (%)	199 (24.9)	141 (50.0)	<.0001
Cardiovascular disease, n (%)	26 (3.3)	17 (6.0)	0.040
Vitamin supplementation, n (%)	154 (19.3)	53 (18.8)	0.860
Exercise, n (%)			
Never	385 (48.3)	128 (45.6)	0.068
Light	112 (14.0)	28 (10.0)	
Regular	301 (37.7)	125 (44.5)	

Data are expressed as means ± S.D.

* Data are expressed as median (1^st^ quartile, 3^rd^ quartile). *P* values are from the t-test using logarithmic transformed values due to skewed distribution.

† Regular: ≥3 times/week, ≥30 minutes per session; light: <3 times/week.

Abbreviations: HOMA-IR, homeostasis model assessment of insulin resistance; NAFLD, non-alcoholic fatty liver disease; LAI, liver attenuation index; 25(OH)D, serum 25-hydroxyvitamin D.

Because vitamin D synthesis requires sun exposure, time of year could be an important factor for serum vitamin D levels. Therefore, when we assessed the associations between 25(OH)D levels and each metabolic variable, age, sex, season, exercise status, and vitamin supplementation were all included in the regression models. Multivariate logistic regression analyses were performed to estimate the odds ratios (ORs) for NAFLD across the month-matched quartiles of 25(OH)D after adjusting for age, sex, exercise, vitamin supplementation, history of ≥7 days of hospitalization during the previous 3 months, active cancer, chronic pulmonary diseases, or cardiovascular diseases, and body mass index or visceral fat (the highest 25(OH)D quartile (Q4) served as a reference group). Because serum vitamin D levels fluctuate throughout the year, we categorized individual patients’ 25(OH)D levels into quartiles by month, then combined the collections of each quartile as month-matched quartiles [19]. However, we obtained similar results using absolute vitamin D levels as when the quartile-based approach for monthly cutoffs was used. All reported *p* values were two-tailed. *P*-values of less than 0.05 were considered statistically significant.

## Results

Table 1 shows the characteristics of the study subjects according to the presence/absence of diabetes. The mean age of total subjects was 56.9±7.3 years old. Subjects with diabetes (N = 282) were older and had worse metabolic profiles than those without diabetes (N = 799). Also, both ALT and AST levels were higher in diabetic subjects than in non-diabetic subjects (ALT and AST, means ± S.D., with vs. without diabetes, 27.6±16.7 vs. 22.8±11.4 and 26.5±10.7 vs. 24.5±6.9, *p*<0.001 and *p* = 0.004, respectively). The prevalence of vitamin D deficiency (25(OH)D <50 nmol/L) and median 25(OH)D levels were not different according to the presence/absence of diabetes. Seventy nine percent of total study subjects had vitamin D deficiency. The prevalence of vitamin D deficiency was lower in subjects with vitamin supplementation than those without [n (%), with vs. without, 131 (63.3%) vs. 725 (83.0%), *p*<0.001]. Among non-diabetic subjects, worse metabolic profiles were observed in the IR+ group than in the IR- group, but 25(OH)D levels were not different according to the presence/absence of insulin resistance (Table S2).

### Associations between 25(OH)D Levels and Metabolic Covariates in Separate Regression Models

**Figure 1:**
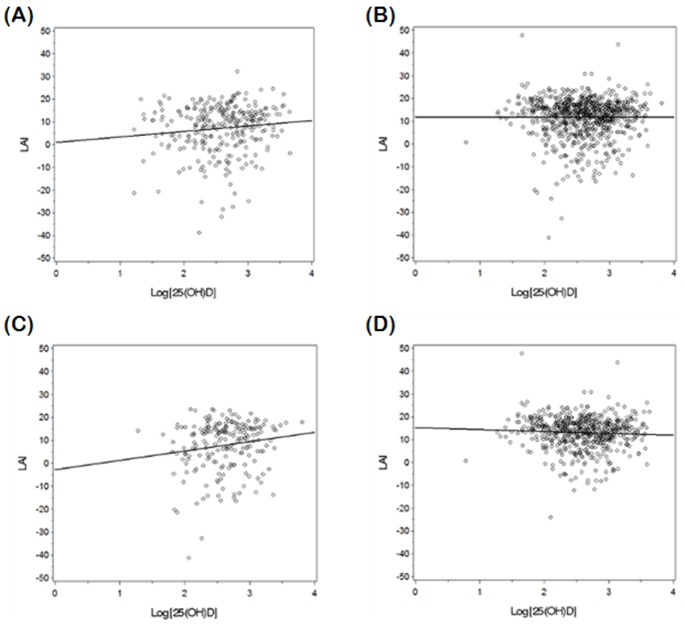
Correlation plots between log-transformed 25(OH)D and liver attenuation index (LAI) in diabetic (A), non-diabetic (B), non-diabetic, IR+ (C) and non-diabetic, IR- (D) subjects, respectively. (correlation coefficient (*r*) and *p* value (*p*) of A, B, C, and D: A, *r = *0.1137, *p* = 0.057; B, *r* = −0.0005, *p* = 0.990; C, *r* = 0.1535, *p* = 0.027; D, *r* = −0.0570, *p* = 0.167).

In the separate regression models for each metabolic variable, 25(OH)D level was negatively associated with waist circumference, fasting insulin, HOMA-IR, triglycerides, ALT, AST levels, and visceral abdominal fat and positively associated with LAI (β = 0.004, *p* = 0.002) among all study participants (n = 1081) after adjusting for age, sex, season, exercise, and vitamin supplementation. The associations between 25(OH)D and metabolic variables, including LAI, in subjects with diabetes were similar to those seen among all study subjects (Table 2, Figure 1A). However, in those without diabetes, only triglyceride level was negatively associated with 25(OH)D and LAI was not associated with 25(OH)D (Table 2, Figure 1B). When we classified subjects without diabetes by insulin resistance status, triglycerides, ALT, AST levels, and LAI levels were associated with 25(OH)D in the IR+ group (Table 2, Figure 1C). Triglycerides levels and visceral abdominal fat were associated with 25(OH)D but LAI was not associated with 25(OH)D in the IR- group (Table 2, Figure 1D).

**Table 2:** Adjusted regression coefficients between log[25(OH)D] level and metabolic variables in separate regression models according to the presence/absence of diabetes or insulin resistance.

	Non-DM (n = 799)				DM (n = 282)	
	IR(−) (n = 592)		IR(+) (n = 207)			
Variables	β	*p-value*	β	*p-value*	β	*p-value*
Waist circumference (cm)	−0.0029	0.300	−0.0042	0.285	−0.0075	0.031
Systolic blood pressure (mmHg)	0.00004	0.978	0.0003	0.866	−0.0010	0.595
Fasting plasma glucose (mmol/L)	0.0333	0.480	0.0328	0.614	−0.0076	0.437
2hr-OGTT plasma glucose(mmol/L)	−0.0025	0.838	−0.0312	0.101	−0.0124	0.270
Fasting insulin (uU/mL)	−0.0178	0.068	−0.0007	0.906	−0.0027	0.008
2hr-OGTT-insulin (uU/mL)	0.0005	0.400	−0.0008	0.145	−0.0004	0.578
HOMA-IR	−0.0652	0.115	−0.0005	0.986	−0.0091	0.001
Triglycerides (mmol/L)	−0.0572	<.001	−0.0797	0.002	−0.0733	0.002
HDL-cholesterol (mmol/L)	−0.0789	0.247	0.1665	0.141	−0.1156	0.378
ALT (IU/L)	−0.0011	0.574	−0.0053	0.004	−0.0021	0.221
AST (IU/L)	−0.0003	0.909	−0.0086	0.005	−0.0026	0.312
Visceral abdominal fat (cm^2^)	−0.0012	0.049	0.0002	0.800	−0.0018	0.006
Liver attenuation index (HU)	0.0015	0.477	0.0059	0.017	0.0062	0.017

Each model was adjusted for age, sex, season, exercise, and vitamin supplementation.

Abbreviations: OGTT, 75-g oral glucose loading test; HOMA-IR, homeostasis model assessment of insulin resistance.

### ORs for NAFLD According to the 25(OH)D Quartiles in Subjects with or without Diabetes and Insulin Resistance

We investigated the ORs for the presence of NAFLD according to 25(OH)D quartiles matched by month. Among all study subjects, the adjusted ORs for NAFLD increased sequentially across decreasing quartiles of 25(OH)D (Table 3). This relationship was more prominent for subjects with diabetes. The lowest 25(OH)D quartile (Q1) had a 3.2-fold increased risk of NAFLD over the highest 25(OH)D quartile (Q4) when adjusting for age, sex, exercise, vitamin supplementation, active cancer, recent hospitalization, chronic pulmonary and cardiovascular diseases, and body mass index. When the model was adjusted for visceral fat area, it was marginally significant. In contrast, there was no increase in ORs for NAFLD by 25(OH)D quartile in subjects without diabetes. However, when we classified subjects without diabetes by insulin resistance, the IR+ Q1 group showed an approximately 3.9-fold increased OR for NAFLD over the IR+ Q4 group, even after additionally adjusting for visceral fat, HbA1c, hypertension, and lipid profile, whereas no significant difference among quartiles was found in the IR− group (Table 3).

**Table 3:** Odds ratios for NAFLD in subjects with or without diabetes according to the month-matched 25(OH)D quartiles.

		Q1			Q2			Q3			Q4	*p for trend*
		OR	(95% CI)		OR	(95% CI)		OR	(95% CI)			
Total	Model1	2.2	1.4	3.5	2.0	1.3	3.2	1.6	1.0	2.5	*ref*	0.001
	Model2	2.1	1.3	3.4	1.9	1.2	3.0	1.6	1.0	2.6	*ref*	0.004
	Model3	1.8	1.1	3.0	1.8	1.1	3.0	1.5	0.9	2.4	*ref*	0.019
	Model4	1.6	1.0	2.7	1.7	1.0	2.8	1.5	0.9	2.4	*ref*	0.063
Non-DM	Model1	1.7	0.9	3.2	1.8	1.0	3.3	2.0	1.1	3.5	*ref*	0.116
	Model2	1.9	1.0	3.6	1.6	0.8	3.0	2.1	1.1	3.8	*ref*	0.133
	Model3	1.6	0.9	3.1	1.6	0.9	3.1	1.9	1.1	3.6	*ref*	0.222
	Model4	1.5	0.8	3.0	1.6	0.8	3.1	2.0	1.1	3.7	*ref*	0.311
DM	Model1	3.1	1.4	7.1	2.9	1.3	6.3	1.9	0.9	4.2	*ref*	0.004
	Model2	3.2	1.3	7.7	2.4	1.0	5.6	1.7	0.7	3.9	*ref*	0.008
	Model3	2.5	1.0	6.2	1.9	0.8	4.5	1.6	0.7	3.8	*ref*	0.051
	Model4	2.0	0.8	5.2	1.6	0.6	3.8	1.4	0.6	3.5	*ref*	0.150
non-DM,IR-	Model1	0.9	0.4	2.2	1.6	0.7	3.4	2.1	1.0	4.3	*ref*	0.712
	Model2	0.9	0.4	2.3	1.7	0.7	3.8	2.3	1.1	4.8	*ref*	0.708
	Model3	0.8	0.3	1.9	1.6	0.7	3.6	2.1	1.0	4.5	*ref*	0.448
	Model4	0.7	0.3	1.8	1.5	0.6	3.4	2.1	1.0	4.5	*ref*	0.352
non-DM,IR+	Model1	3.8	1.4	10.3	2.5	0.9	6.8	2.2	0.8	5.7	*ref*	0.011
	Model2	3.8	1.3	10.6	2.1	0.7	6.1	1.8	0.7	4.9	*ref*	0.015
	Model3	3.8	1.4	10.3	2.5	0.9	7.2	1.9	0.7	5.1	*ref*	0.010
	Model4	3.9	1.3	11.3	2.9	1.0	8.5	2.2	0.8	6.2	*ref*	0.013

Model 1: adjusted for age, sex, exercise, vitamin supplementation, active cancer, recent hospitalization, chronic pulmonary diseases, and cardiovascular diseases.

Model 2: model 1+ body mass index.

Model 3: model 1+ visceral abdominal fat.

Model 4: model 3+HbA1c, hypertension, total cholesterol, triglycerides, HDL-cholesterol.

Abbreviations: non-DM, IR-, non-diabetic subjects with HOMA-IR <2.5; non-DM, IR+, non-diabetic subjects with HOMA-IR ≥2.5; Q1∼Q4, month-matched 25(OH)D quartiles 1∼4.

## Discussion

In this study, we demonstrated that NAFLD was closely associated with low circulating vitamin D levels in Korean adults with diabetes or insulin resistance. Although a few studies have reported that 25(OH)D levels are inversely correlated with NAFLD in different ethnicities, either the number of study subjects was small [6] or the visceral obesity-independent effect of vitamin D deficiency on NAFLD was not evaluated [20], [21]. To the best of our knowledge, this is the first study to evaluate the correlation of vitamin D levels with NAFLD according to insulin resistance and diabetes status, regardless of visceral obesity, in a large population-based study.

There are several lines of evidence demonstrating an association between vitamin D and liver disease. The liver is a key organ in vitamin metabolism, as it transforms vitamin D into 25(OH)D and accelerates uptake of vitamin D by producing bile acid. The presence of the vitamin D receptor in liver cells suggests a role of vitamin D in liver pathophysiology [22]. In several human studies, vitamin D receptor polymorphism is associated with hepatic disorders such as primary biliary cirrhosis and autoimmune hepatitis [23], [24]. However, to date, experimental evidence regarding the role of vitamin D in the development or treatment of NAFLD has been limited. Recently, Nakano et al. reported the effects of phototherapy or active vitamin D treatment on non-alcoholic steatohepatitis in a rat model, in part due to increased gene expression of adiponectin and its receptor [7]. Another study using an obese rat model showed that vitamin D deficiency exacerbates NAFLD through an increase of hepatic resistin and Toll-like receptor activation [11].

Visceral obesity is known to be associated with low vitamin D status regardless of body mass index [25]. As visceral obesity and NAFLD are highly correlated, it is expected that the association between vitamin D insufficiency and NAFLD may be mediated by visceral obesity. However, NAFLD is often independent of visceral fat-derived insulin resistance [26]. Patients with severe lipodystrophy manifest severe hepatic steatosis and hepatic insulin resistance, but exhibit a lack of visceral fat [27]. Fabbrini et al. demonstrated that intrahepatic fat content, not visceral fat amount, is a better marker of the insulin sensitivity and metabolic derangements associated with obesity [28]. In addition, a reduction in visceral fat did not improve hepatic insulin resistance in obese individuals [29]. Taken together, these studies demonstrate that ectopic lipid accumulation within the liver can specifically cause hepatic insulin resistance independent of visceral obesity. The present study showed an association between vitamin D deficiency and NAFLD independent of visceral obesity. This suggests that vitamin D deficiency may be specifically associated with hepatic lipid accumulation and hepatic insulin resistance regardless of visceral fat accumulation, although the mechanism cannot be determined from the present study.

We demonstrated a relationship between NAFLD and vitamin D deficiency only in subjects with diabetes or insulin resistance. Similarly, the negative association between HOMA-IR and vitamin D levels was present only in those with diabetes. In accordance with the results of this study, the association between vitamin D deficiency and NAFLD was more prominent in rats that were fed a high-fat diet and had greater insulin resistance than in those fed a low-fat diet in a previous study [11]. Although a few studies have investigated the impact of vitamin D deficiency according to diabetes or insulin resistance status, the association between hypovitaminosis D and metabolic variables was exhibited only in subjects with diabetes in a Saudi population [10]. In previous studies, the association between 25(OH)D levels and insulin resistance varied according to the characteristics of study subjects such as ethnicity, sex, obesity, or menopausal status [30], [31], [32], [33]. A significant negative association between 25(OH)D levels and HOMA-IR was observed in non-diabetic adults in the Framingham Offspring Study [34]. In a nationally representative sample of U.S. adults, the inverse association between 25(OH)D and metabolic disturbance (i.e. fasting blood glucose, HOMA-IR, C-reactive protein) was significantly stronger among subjects with central obesity than those without [30]. Although trials have yielded conflicting results, recent studies have shown that vitamin D supplementation reduces insulin resistance in subjects with insulin resistance and vitamin D deficiency [35], [36], but this effect was not demonstrated in those without insulin resistance [37], [38]. These studies suggest that the impact of vitamin D deficiency on metabolic derangement may be greater in people with diabetes or insulin resistance than in those without. Therefore, we investigated whether the association between vitamin D and NAFLD differed according to the status of diabetes or insulin resistance. We used a cutoff point of HOMA-IR defining insulin resistance as the 75th percentile value as suggested in previous studies [18], [39]. A previous Korean study reported a cutoff value of HOMA-IR for increased risk of metabolic syndrome in healthy adults of 2.56 [40]. In our study population, the 75^th^ percentile of HOMA-IR value in non-diabetic subjects was about 2.5 in both sexes. Using the current 75^th^ percentile of HOMA value (2.5) in non-diabetic subjects, the metabolic and anthropometric characteristics were well contrasted between subjects with or without insulin resistance.

It is difficult to verify the cause of the discrepant relationship between vitamin D and NAFLD according to diabetes or insulin resistance status in the present study. One possible mechanism for this different association is the role of fibroblast growth factor (FGF)19, which has been shown to be a novel regulator of hepatic lipid synthesis [41]. Transgenic expression of the human FGF19 gene in obese/diabetic mice was shown to cause an increase in energy expenditure and reversed hepatic steatosis, hyperlipidemia, and diabetes [42], [43]. Because vitamin D could induce the expression of FGF19 [44], some metabolic effects of vitamin D may be mediated by FGF19. However, recent evidence has shown that the hepatic response to FGF19 was impaired in NAFLD patients with a HOMA score ≥2.5 in contrast to a normal response in NAFLD patients with a HOMA score <2.5 [45]. Interestingly, we also observed an association between 25(OH)D levels and NAFLD only in subjects with a HOMA score ≥2.5. We can hypothesize that the impaired metabolic action of FGF19 in the insulin resistant state may accentuate the effect of low vitamin D concentration on NAFLD, although this has not yet been proven. A second possible hypothesis is the potential stimulatory effect of vitamin D on adiponectin expression. Recent studies in type 2 diabetes have demonstrated an association between vitamin D deficiency and low adiponectin level [10] and an improvement of adiponectin levels after vitamin D therapy in type 2 diabetic patients [46]. A potential mechanism is the inhibitory effect of vitamin D on the renin angiotensin system (RAS), which leads to inhibition of adiponectin secretion [47]. In other words, vitamin D can increase adiponectin concentrations via inhibition of RAS. As adiponectin is known as a protective molecule in the development of NAFLD [48], [49], the lower adiponectin concentrations [50], [51] associated with vitamin D deficiency in subjects with diabetes or insulin resistance may be responsible for the development of NAFLD in those with high metabolic risk.

Given that millions of people are vitamin D deficient and that there is a strong association between vitamin D deficiency and NAFLD in subjects with insulin resistance or diabetes, vitamin D supplementation could be a potential tool to prevent the progression of NAFLD in metabolically high-risk individuals. Alternatively, improvement in NAFLD may restore circulatory vitamin D levels. In either case, this relationship deserves careful examination because of the importance of vitamin D deficiency in health promotion and public health policy-making.

This study has several limitations. First, the causal relationship between vitamin D and NAFLD could not be investigated in this cross-sectional study. Second, we diagnosed NAFLD based on low LAI using computed tomography without histological confirmation. However, computed tomography could be a reasonable, less-invasive alternative to liver biopsy in epidemiologic studies of the general population [52]. Finally, we did not assess the exact amount of vitamin D intake or exposure to ultraviolet B radiation, which could affect circulating vitamin D levels, although including vitamin supplementation and season in the regression models did not substantially alter the results.

In conclusion, low vitamin D status was associated with NAFLD in the Korean adult population, especially in subjects with insulin resistance or diabetes, irrespective of visceral obesity. Whether vitamin D supplementation could improve metabolic derangement in subjects with insulin resistance or diabetes should be explored in future randomized prospective trials.

## Supporting Information

Table S1 **Comparison of the characteristics of subjects enrolled in the present study and those who were excluded (the remainder).**
(DOC)

Table S2 **Characteristics of study subjects according to the presence or absence of insulin resistance in subjects without diabetes.**
(DOC)
