# Supplementary material for: Low Vitamin D Status Is Associated with Nonalcoholic Fatty Liver Disease Independent of Visceral Obesity in Korean Adults
Source: PLoS One. 2013 Oct 9;8(10):e75197. doi: 10.1371/journal.pone.0075197 (PMC3793981; doi:10.1371/journal.pone.0075197)
Supplement: Table S1 — Comparison of the characteristics of subjects enrolled in the present study and those who were excluded (the remainder). (DOC) [file pone.0075197.s001.doc]

Table S1. Comparison of the characteristics of subjects enrolled in the present study and those who were excluded (the remainder)

| Variables | people included in this study (n=1081) | †the remainder (n=1495) | *p-value* |
| --- | --- | --- | --- |
| Age (years) | 56.9 ± 7.3 | 56.9 ± 7.4 | 0.994 |
| Men, n(%) | 344 (31.8) | 491 (32.8) | 0.585 |
| Waist circumference (cm) | 80.5 ± 8.2 | 80.3 ± 8.1 | 0.483 |
| Body mass index (kg/m^2^) | 24.7 ± 2.9 | 24.6 ± 2.9 | 0.475 |
| Systolic blood pressure (mmHg) | 113.6 ± 14.7 | 113.8 ± 15.5 | 0.742 |
| Fasting plasma glucose (mmol/L) | 5.6 ± 1.7 | 5.6 ± 1.7 | 0.974 |
| Fasting insulin* (IU/L) | 9.0 (7.0,11.5) | 8.6 (6.7,11.1) | 0.032 |
| HOMA-IR* | 2.1 (1.6,2.8) | 2.0 (1.5,2.7) | 0.082 |
| Triglycerides* (mmol/L) | 1.40 (0.99,2.00) | 1.36 (0.97,1.95) | 0.146 |
| HDL-cholesterol (mmol/L) | 1.10 ± 0.27 | 1.13 ± 0.28 | 0.006 |
| ALT* (IU/L) | 20.0 (16.0,27.0) | 20.0 (16.0,27.0) | 0.297 |
| AST* (IU/L) | 23.0 (21.0,27.0) | 23.0 (20.0,27.0) | 0.247 |
| Visceral abdominal fat (cm2) | 82.7 ± 37.1 | 80.3 ± 36.4 | 0.101 |
| Liver attenuation index (HU) | 10.6 ± 10.2 | 10.4 ± 9.6 | 0.640 |
| NAFLD [LAI<5, n(%)] | 229 (21.2) | 312 (20.9) | 0.847 |
| Hypertension, n(%) | 340 (31.5) | 480 (32.1) | 0.725 |
| Cardiovascular disease, n(%) | 43 (4.0) | 80 (5.4) | 0.107 |
| Exercise, n(%) |  |  | 0.067 |
| Never | 513 (47.5) | 756 (50.6) |  |
| Light | 140 (13.0) | 214 (14.3) |  |
| Regular | 426 (39.5) | 523 (35.0) |  |

* median (1^st^ quartile, 3^rd^ quartile)

† the remainder: among the participants in the fifth biennial examination of KoGES, subjects without 25(OH)D level measurements were not included in the present study.
