# Supplementary material for: Low Vitamin D Status Is Associated with Nonalcoholic Fatty Liver Disease Independent of Visceral Obesity in Korean Adults
Source: PLoS One. 2013 Oct 9;8(10):e75197. doi: 10.1371/journal.pone.0075197 (PMC3793981; doi:10.1371/journal.pone.0075197)
Supplement: Table S2 — Characteristics of study subjects according to the presence or absence of insulin resistance in subjects without diabetes. (DOC) [file pone.0075197.s002.doc]

Table S2. Characteristics of study subjects according to the presence or absence of insulin resistance in subjects without diabetes

| Variables | IR (-) (n=592) | IR (+) (n=207) | *p-value* |
| --- | --- | --- | --- |
| Age (years) | 55.7 ± 6.8 | 56.2 ± 6.5 | 0.333 |
| Men, n(%) | 176 (29.7) | 64 (30.9) | 0.748 |
| Waist circumference (cm) | 77.9 ± 7.3 | 83.9 ± 7.4 | <.0001 |
| Body mass index (kg/m^2^) | 23.9 ± 2.6 | 25.9 ± 2.8 | <.0001 |
| Systolic blood pressure (mmHg) | 112.2 ± 14.4 | 115.0 ± 14.2 | 0.013 |
| Fasting plasma glucose (mmol/L) | 5.0 ± 0.4 | 5.4 ± 0.4 | <.0001 |
| Fasting insulin^*^ (uU/mL) | 7.5 (6.2,9.0) | 12.8 (11.5,15.5) | <.0001 |
| HOMA-IR^*^ | 1.7 (1.4,2.0) | 3.0 (2.7,3.7) | <.0001 |
| Triglycerides^*^ (mmol/L) | 1. 3 (0.9,1.7) | 1.7 (1.2,2.4) | <.0001 |
| HDL-cholesterol (mmol/L) | 1.2 ± 0.3 | 1.0 ± 0.2 | <.0001 |
| ALT^*^(IU/L) | 19.0 (15.0,23.0) | 24.0 (18.0,33.0) | <.0001 |
| AST^*^(IU/L) | 23.0 (20.0,26.0) | 24.0 (21.0,29.0) | <.0001 |
| 25(OH)D* (nmol/L) | 33.9 (24.2,47.6) | 34.7 (26.2,46.6) | 0.323 |
| Visceral abdominal fat (cm^2^) | 70.3 ± 30.8 | 97.4 ± 33.0 | <.0001 |
| Liver attenuation index (HU) | 13.2 ± 8.9 | 7.9 ± 10.9 | <.0001 |
| NAFLD [LAI<5, n(%)] | 69 (11.7) | 62 (30.0) | <.0001 |
| Vitamin D deficiency [25(OH)D<50nmol/L, n(%)] | 469 (79.2) | 169 (81.6) | 0.455 |
| Hypertension, n(%) | 133 (22.5) | 66 (31.9) | 0.007 |
| Cardiovascular disease, n(%) | 19 (3.2) | 7 (3.4) | 0.904 |
| Vitamin supplementation, n(%) | 123 (20.8) | 31 (15.0) | 0.069 |
| Exercise, n(%) |  |  |  |
| Never | 272 (46.0) | 113 (54.9) | 0.080 |
| Light | 85 (14.4) | 27 (13.1) |  |
| Regular | 235 (39.7) | 66 (32.0) |  |

* median (1^st^ quartile, 3^rd^ quartile)

†Regular: ≥3 times/week, ≥30 minutes per session; light: <3 times/week

Abbreviations: HOMA-IR, homeostasis model assessment of insulin resistance; NAFLD, non-alcoholic fatty liver disease; LAI, liver attenuation index; 25(OH)D, serum 25-hydroxyvitamin D
